# Quantitative Native Proteomics by Capillary Zone Electrophoresis-Mass Spectrometry

**DOI:** 10.1021/acs.analchem.5c06099

**Published:** 2025-11-12

**Authors:** Fei Fang, Liangliang Sun

**Affiliations:** Department of Chemistry, 3078 Michigan State University, 578 S. Shaw Lane, East Lansing, Michigan 48824, United States

## Abstract

Accurately measuring complexoform dynamics (i.e., composition and/or abundance changes) in cells is vital for advancing fundamental and translational research. In this work, we present a pilot study establishing capillary zone electrophoresis (CZE)-mass spectrometry (MS)-based quantitative native proteomics to determine significant changes in complexoform abundance during the transition from logarithmic to stationary phase growth in *Escherichia coli*. The approach integrates (1) efficient and fast native CZE-MS to obtain the mass and signal intensity of complexoforms for label-free quantification, (2) in-source collision-induced dissociation, enabling informative fragmentation that reveals oligomeric states, and (3) denatured top-down proteomics for the identification of proteoforms, which form the complexoforms. We revealed differentially expressed complexoforms during the growth of *Escherichia coli*. For example, the glutamate decarboxylase beta hexamer (∼317 kDa) exhibits a significantly higher abundance at the stationary phase, which aligns with its biological function. This work represents the first quantitative native proteomics study using online native CZE-MS.

## Graphical abstract

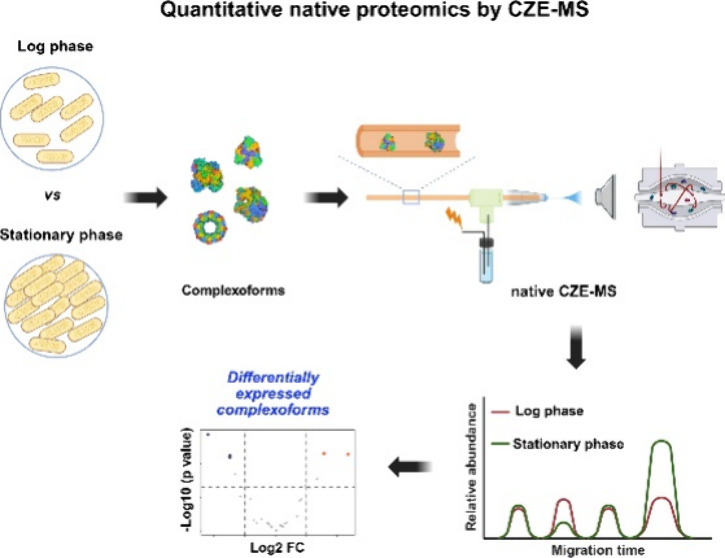

In living cells, most proteins form stable or transient functional assemblies, called complexoforms,^1^ that regulate essential processes such as the cell cycle, metabolism, and signal transduction.^2–5^ Native proteomics aims to measure the endogenous complexoforms in cells, tissues, and biological fluids, in discovery mode and on a proteome scale.^6–8^ While quantitative bottom-up proteomics^9–11^ and top-down proteomics^12–15^ have been widely used to determine protein/proteoform abundance changes across various biological conditions, very few quantitative native proteomics studies have been performed to determine the abundance change of endogenous complexoforms in cells in different conditions.

Native mass spectrometry (nMS), which provides essential insights into the structures, functions, and dynamics of proteoforms and complexoforms near physiological conditions, has emerged as a powerful tool for complexoform analysis.^16–19^ In combination with offline native size-exclusion chromatography separation, direct infusion, and high-field asymmetric waveform ion mobility spectrometry (FAIMS) separation, Fabio et al. performed native proteomics of breast cancer cells and epidermal growth factor receptor-overexpressed breast cancer cells, and identified more than 100 complexoforms from 17 protein complexes (≤70 kDa) in the breast cancer cells.^20^ The Ge group applied native proteomics to study endogenous complexoforms in human heart tissues with automated, online interfacing of size-exclusion and mixed-bed ion-exchange chromatography, detecting 133 native proteoforms and endogenous complexoforms (up to 350 kDa).^21^

Due to the high separation efficiency and high detection sensitivity for complexoforms, native capillary zone electrophoresis-MS (nCZE-MS) has been applied to analyzing complexoforms in low-complexity protein samples.^22–25^ Our group published the first example of native proteomics of a complex biological sample using online nCZE-MS, with the identification of 23 complexoforms smaller than 30 kDa.^7^ In 2024, we detected 72 complexoforms from a whole *E. coli* cell lysate covering a mass range of 30–400 kDa in a single nCZE-MS run using an ultrahigh-mass-range (UHMR) Orbitrap while consuming only 50-ng protein material.^26^

In this work, we represent the first example of label-free quantitative native proteomics using nCZE-MS for a complex biological sample, determining the differentially expressed complexoforms during the transition from logarithmic (log) to stationary phase growth (Figure 1). Once *E. coli* cells adapt to the new cultivation conditions, they begin to divide exponentially, entering the log phase of growth. As nutrients in the medium become depleted, the bacterial culture subsequently enters the stationary phase, where its internal systems of protection against stress become activated in response to harsh environmental influences.^27,28^ The *E. coli* cells in the stationary phase can dramatically change their organization both at the molecular and cellular levels, achieving orders of magnitude more resistance to antimicrobials and acquiring the ability to survive even under extremely adverse environmental settings.^29^ Hence, elucidating the regulatory mechanisms mediated by distinct complexoforms during the stationary phase is crucial for advancing both fundamental knowledge and practical applications.

**Figure 1:**
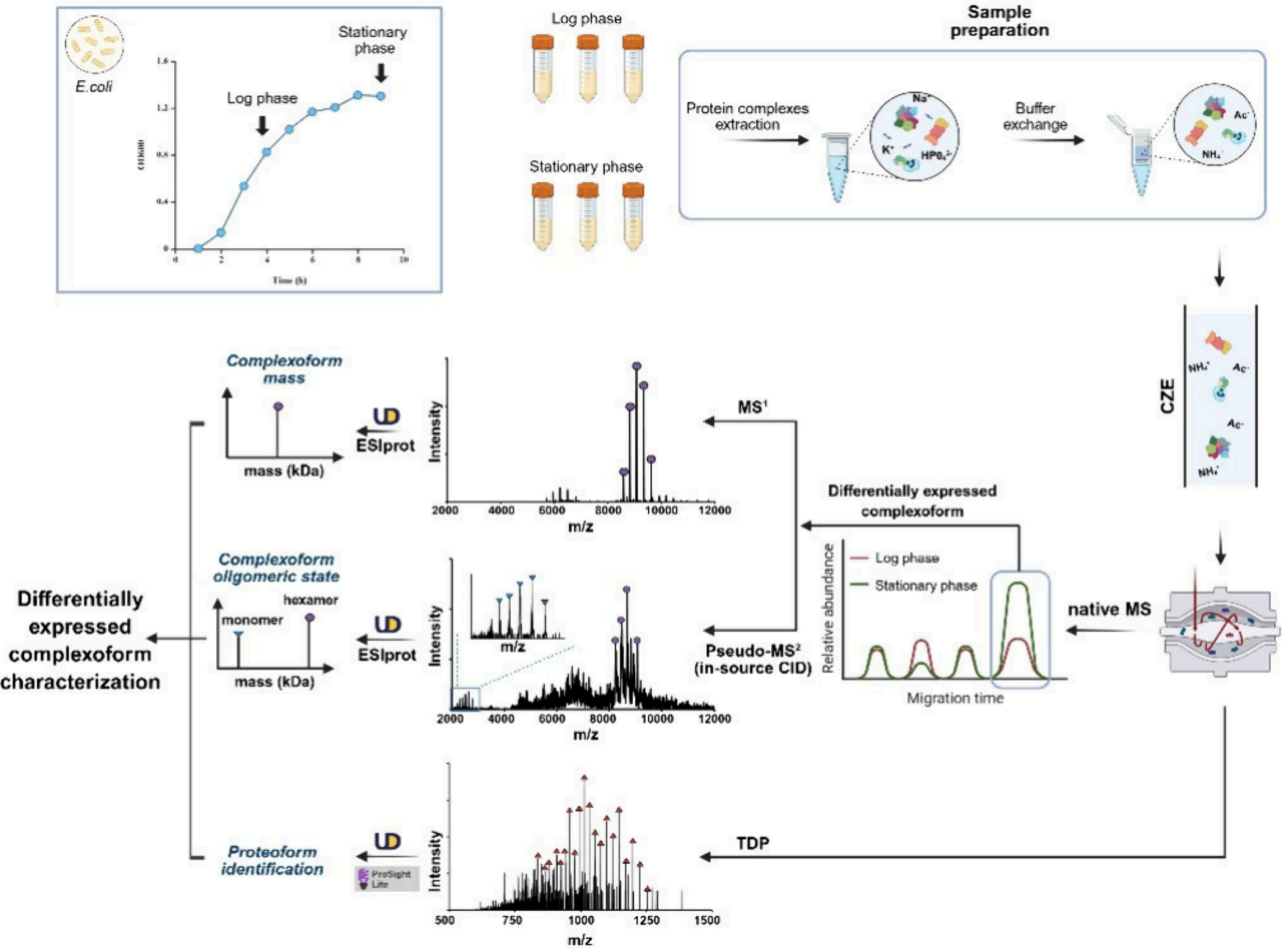
Flowchart of nCZE-MS for quantitative native proteomics of *E. coli* cell lysate from log and stationary phases. The figure is created using BioRender and is used here with permission.

Briefly, the whole *E. coli* cell lysates from two growth phases were extracted with Dulbecco’s phosphate-buffered saline (DPBS) buffer containing complete protease inhibitors and phosphatase inhibitors. The cell lysate was buffer-exchanged on an Amicon-10 kDa centrifugal filter unit to a buffer containing 50 mM ammonium acetate (NH_4_Ac, pH 6.9) and subjected to nCZE-MS. The online nCZE-MS was assembled by coupling a Sciex CESI-8000 Plus capillary electrophoresis (CE) autosampler to a Thermo Fisher Scientific Q-Exactive UHMR mass spectrometer through a commercialized electrokinetically pumped sheath flow CE-MS interface (EMASS-II, CMP Scientific).^30,31^ To reduce the protein nonspecific adsorption onto the capillary inner wall, a 90 cm-long capillary with neutral coating (i.e., linear polyacrylamide, LPA) was employed for the nCZE separation. The background electrolyte (BGE) and sheath buffer used for nCZE were 25 mM NH_4_Ac (pH ∼ 7.0) and 10 mM NH_4_Ac (pH ∼ 7.0), respectively. To determine the number of detected native proteoforms, the raw MS data were split into 30-s windows, followed by mass deconvolution using UniDec and ESIprot.^32,33^ With precursor ion peaks corresponding to each complexoform extracted from the MS^1^ spectrum across runs, the resulting intensities were compared to quantify and determine the differentially expressed complexoforms.

To further identify the detected differentially expressed complexoforms, the same samples were subjected to nCZE-MS with in-source collisional induced dissociation (CID) fragmentation, which generated pseudo-MS^2^ spectra by causing subunit dissociation through the unfolding or elongation and subsequent ejection of a highly charged monomer.^34^ The obtained spectra were deconvoluted using UniDec and ESIprot, with the masses of monomer and corresponding complexoform employed, to determine the oligomeric state of each complexoform.

In addition, denaturing top-down proteomics (TDP) was applied to identify the high-abundance proteoforms from the cell lysate. Following MS^1^ spectral deconvolution with UniDec, proteoform candidates were determined by matching the observed masses with those listed in UniProt. Subsequently, the processed MS^2^ spectra were analyzed with ProSight Lite^35^ against the sequences of the proteoform candidates to assign the corresponding proteoform.

By integrating the above intact complexoform mass (MS^1^), oligomeric state of complexoforms (pseudo-MS^2^), as well as corresponding monomer mass and sequence information (denaturing TDP), the complexoforms differentially expressed during bacterial growth were characterized. The detailed experimental procedure is described in the Supporting Information.

To assess the repeatability of nCZE-MS for complex samples, the whole cell lysates extracted from *E. coli* cells in the log and stationary phases were subjected to nCZE-MS in biological triplicate, respectively. As shown in Figure S1A, complexoforms can be efficiently extracted and separated using CZE-MS under native conditions. A consistent decrease in migration time in the second and third runs was observed compared to the first run, most likely due to changes at the capillary inner wall after the first run of the *E. coli* sample. However, after migration time alignment, we discovered a high reproducibility in the migration time and peak intensity of selected proteoforms/complexoforms separated with nCZE (Table S1), with relative standard deviations (RSDs) of migration time between 0.94%–3.2% and peak intensity between 12%–27%, which is similar to the result obtained by simple protein complexes separated under native conditions.^36^ Figure S1B shows the extracted ion electropherograms of the selected proteoforms/complexoforms, which are much wider than those for denaturing CZE, possibly due to the protein dispersion under the applied pressure and nonspecific protein adsorption on the capillary inner wall. shows the mass spectra with labeled charges and masses of the selected proteoforms/complexoforms. The above results demonstrate the reasonable reproducibility and robustness of the nCZE-MS method, enabling confident label-free quantification.

After spectrum averaging and mass deconvolution, complexoforms ranging from 20 kDa to 320 kDa were detected in the *E. coli* sample. Totally, we quantified 33 proteoforms or complexoforms from the cell lysate, among which 4 exhibited statistically significant abundance change, Figure 2A and Figure S2. The list of quantified proteoforms/complexoforms is shown in Supporting Information Excel spreadsheet. The expression level of three large complexoforms, whose masses are 88 kDa, 209 kDa, and 211 kDa, dramatically decreased during growth. Interestingly, a 317 kDa complexoform was identified as being significantly upregulated during the stationary phase compared with the log phase (Figure 2A and B), which might play an important role in *E. coli* growth. The MS^1^ and pseudo-MS^2^ results showed that the molecular weight of the intact protein complex is around 317 449 Da, while the released monomer upon collisional activation is 52 895 Da, Figure 2C and D, illustrating that this complexoform is a homohexamer.

**Figure 2:**
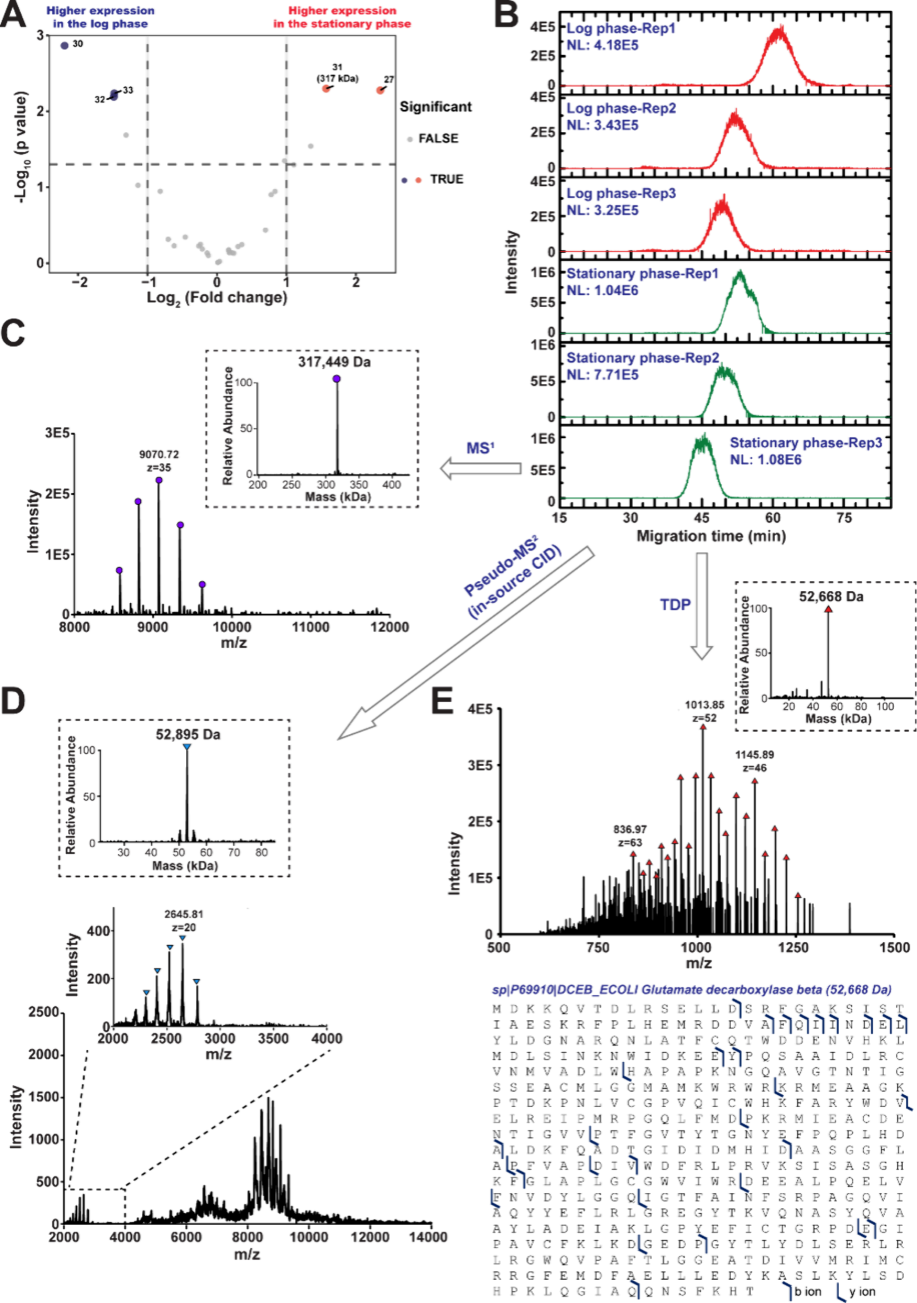
Characterization of the complexoforms that is differentially expressed during the two phases of *E. coli* growth. (A) Volcano plot showing differentially expressed complexoforms between the *E. coli* cells in the two growth phases. Blue dots and red dots represent complexoforms having statistically significantly higher abundance in the log and stationary phases, respectively. The numbers represent the number of features listed in the Supporting Information Excel spreadsheet. The molecular weight of the differentially expressed complexoform is labeled. (B) The electropherograms of the complexoform (317 kDa) extracted from whole *E. coli* cell lysate in the log and stationary phase in biological triplicates, respectively. Mass spectrum and deconvoluted masses of complexoform (317 kDa) with (C) MS^1^, (D) pseudo-MS^2^ (in-source CID), and (E) denaturing top-down proteomics approaches. The sequence and fragmentation patterns of the detected proteoforms are shown.

We also subjected the sample to denaturing top-down proteomics analysis and identified a proteoform with a mass of 52 668 Da, which closely matches the monomer mass of the 317 kDa complexoform observed in the pseudo-MS^2^ spectrum. Our hypothesis here is that the proteoform forming the 317-kDa complexoform is one of the most abundant proteins in the sample, considering that the complexoform showed the strongest signal in the native CZE-MS runs. By searching against UniProt, glutamate decarboxylase beta (GadB) was identified as the proteoform candidate. GadB is a homohexamer composed of six identical subunits, each with a theoretical mass of 52,668.13 Da. By matching the fragment ions using ProSight Lite, the mass difference between the theoretical and observed mass is 2.4 ppm, with 40 fragment ions matched (Figure 2E).

It is noticed that there’s a mass shift (52895–52668 = 227 Da) between the monomers obtained from pseudo-MS^2^ and the TDP result, which might come from the pyridoxal phosphate (PLP) modification (229 Da) on K276 of GadB. As PLP is the cofactor of GadB, the addition of PLP can significantly enhance the activity and stability of this enzyme.^37^ While the noncovalent protein–cofactor interaction is preserved under in-source CID conditions, the PLP information was lost during denaturing top-down proteomics, which causes the mass shift. Consistent with previous Northern and Western blot findings showing increased GadB expression during the stationary phase compared with the exponential phase,^38,39^ the 317 kDa complexoform was identified as GadB with high confidence. Acid resistance (AR) in *E. coli* is defined as the ability to withstand an acid challenge of pH 2.5 or less and is a trait generally restricted to stationary-phase cells.^40^ The GadB is a component of the GAD system, which is the most effective system of AR found in bacteria able to survive in extreme acidic conditions, protecting the stationary-phase cell under naturally occurring acidic environments.^41^

In summary, we demonstrate for the first time that nCZE-MS is an effective platform for quantitative native proteomics of complex proteome samples. Using this approach, 33 large proteoforms/complexoforms were successfully quantified across two main growth phases for *E. coli*. Furthermore, by integrating in-source CID with denaturing top-down proteomics, the method enabled the identification of differentially expressed complexoforms, including GadB—a 317 kDa hexamer composed of six identical subunits—confidently identified as upregulated during *E. coli* growth. For better separation peak capacity and reproducibility, procedures to clean up the capillary inner wall between nCZE-MS runs and improve the capillary inner wall coating through different chemistries, e.g., carbohydrate-based neutral coating,^42^ could be employed to reduce protein adsorption. This work suggests that nCZE-MS is ready for quantitative native proteomics to determine differentially expressed endogenous complexoforms in various biological samples.

## Supplementary Material





## Data Availability

The MS raw files have been deposited to the ProteomeXchange Consortium via the PRIDE partner repository [Perez-Riverol, Y.; Bai, J.; Bandla, C.; García-Seisdedos, D.; Hewapathirana, S.; Kamatchinathan, S.; Kundu, D. J.; Prakash, A.; Frericks-Zipper, A.; Eisenacher, M.; Walzer, M.; Wang, S.; Brazma, A.; Vizcaíno, J. A. The PRIDE Database Resources in 2022: A Hub for Mass Spectrometry-Based Proteomics Evidences. Nucleic Acids Res 2022, 50 (D1), D543–D552. https://academic.oup.com/nar/article/50/D1/D543/6415112] with the data set identifier PXD068962.

## Notes

Experimental section; representative nCZE-MS analysis of endogenous complexoforms from *E. coli* cell lysate (Figure S1); box plots of relative abundance for significantly altered complexoforms obtained from Figure 2A (Figure S2); repeatability of migration time and peak intensity for selected complexoform peaks (Table S1) (PDF)
List of proteoforms/complexoforms quantified from nCZE-MS analysis of *E. coli* cell lysates from log and stationary phases (XLSX)
