# Supplementary material for: Quantitative Native Proteomics by Capillary Zone Electrophoresis-Mass Spectrometry
Source: Anal Chem. 2025 Nov 12;97(46):25385–90. doi: 10.1021/acs.analchem.5c06099 (PMC12658865; doi:10.1021/acs.analchem.5c06099)
Supplement: Supplementary file 1 [file ac5c06099_si_001.pdf]

Supporting Information I

Quantitative Native Proteomics by Capillary Zone Electrophoresis-Mass Spectrometry

Fei Fang and Liangliang Sun*

Affiliations

Department of Chemistry, Michigan State University, 578 S Shaw Lane, East Lansing, MI 48824, United States.

* Corresponding author. lsun@chemistry.msu.edu

Table of Contents

Experimental section……..…....……..…....………..…....…………..…..…....……..…....……..S3

Figure S1. Representative nCZE-MS analysis of endogenous complexoforms from *E. coli* cell lysate. ……..…....……..…....……..…....……..…....……..…....………....…....……..…....……....S8

Figure S2. Box plots of relative abundance for significantly altered complexoforms obtained from Figure 2A. ……..…....……..…....……....……....……....……....……....……....……..…....……....S9

Table S1. The repeatability of migration time and peak intensity was evaluated for selected complexoform peaks (corresponding to those shown in Figure S1) in the *E. coli* stationary phase sample using nCZE-MS, with migration times aligned to the most abundant peak.…....……..… …....……..…..……....……..…..……....……..…..……....……..…..…….S10

References……..…....……..…....………..…....…………..…..…....…………....……..…....…..S11

Experimental section

Materials and chemicals

All reagents, such as Dulbecco's phosphate-buffered saline (DPBS) and Amicon-10 kDa centrifugal filter unit, were purchased from Sigma-Aldrich (St. Louis, MO) unless stated otherwise. Bare fused silica capillaries (50-µm i.d., 360-µm o.d.) were purchased from Polymicro Technologies (Phoenix, AZ). LC/MS grade water, methanol, and ammonium acetate (NH_4Ac), as well as hydrofluoric acid (HF), were purchased from Fisher Scientific (Pittsburgh, PA). Acrylamide was purchased from Acros Organics (Fair Lawn, NJ). Protease inhibitors (cOmplete ULTRA Tables) and phosphatase inhibitors (PhosSTOP) were from Roche (Indianapolis, IN).

Sample preparation

E. coli (strain MG1655) was cultured in Terrific Broth (TB) medium at 37 °C until OD600 reached 0.8 (logarithmic phase) or 1.3 (stationary phase). Each stage was collected in triplicate. After being washed with DPBS three times, approximately 0.4 g pellet was suspended in 3 mL cold DPBS buffer plus complete protease inhibitors and phosphatase inhibitors and homogenized for 30 s, followed by sonication with a Branson Sonifier 250 (VWR Scientific, Batavia, IL) on ice for 1 minute, 15 times. After centrifugation at 20,000 g for 20 minutes at 4 °C, the supernatant containing the extracted proteins was collected. A small aliquot of the diluted sample was used for the bicinchoninic acid (BCA) assay to determine the protein concentration.

For each sample, 200 µg of protein was transferred to an Amicon-10 kDa centrifugal filter unit. The filters were washed with 50 mM NH_4Ac (pH 6.9) twice for buffer exchange, followed by adding 40 µL of 50 mM NH_4Ac (pH 6.9) into each filter unit to extract the proteins on the membrane. The filter units were gently vortexed for 5 min, and the protein solution was taken from the filter units for concentration measurement. The sample concentration was adjusted to ~5mg/mL followed by capillary zone electrophoresis-mass spectrometry (CZE-MS) analysis.

Preparation of linear polyacrylamide (LPA)-coated separation capillary

The inner wall of the separation capillary (50-µm i.d., 360-µm o.d.) was coated with LPA based on the protocol described in previous references[1,2]. Briefly, a bare fused silica capillary was successively flushed with 1 M sodium hydroxide, water, 1 M hydrochloric acid, water, and

methanol, followed by treatment with 50% (v/v) 3-(trimethoxysilyl) propyl methacrylate in methanol for at least 24 hours to introduce carbon-carbon double bonds on the inner wall of the capillary. The treated capillary was filled with degassed acrylamide solution in water (4%) containing ammonium persulfate, followed by incubation at 50 °C water bath for 1h with both ends sealed by silica rubber. After that, the capillary was flushed with water to remove the unreacted reagents. Then, one end of the LPA-coated capillary was etched with HF for 70 minutes to reduce its outer diameter to around 70 µm. A 90 cm LPA-coated capillary (50-µm i.d. and 360-µm o.d.) was used for all CZE-based experiments.

Native CZE-MS

For the CZE-MS-based native quantification experiment, a Beckman CESI8000 Plus capillary electrophoresis autosampler (SCIEX, Framingham, MA) was used for the automated operation of CZE. A commercialized electrokinetically pumped sheath flow interface (EMASS-II, CMP Scientific, Brooklyn, NY) was used to couple CZE to a mass spectrometer[3]. A Q-Exactive UHMR mass spectrometer (Thermo Fisher Scientific, Waltham, MA) was used for the experiments. The interface was directly attached to the mass spectrometer. The electrospray ionization (ESI) emitters of the interface were pulled from borosilicate glass capillaries (1.0 mm o.d., 0.75 mm i.d.) with a Sutter P-1000 flaming/brown micropipet puller (Sutter Instrument, Novato, CA) with an orifice size of ~25 µm. The sheath liquid contains 10 mM NH_4Ac. Voltage for ESI was ~2 kV. The background electrolyte (BGE) for CZE was 25 mM NH_4Ac (pH ~ 7.0).

The transfer capillary temperature was 250 ^oC, and the S-lens RF level was 200. The number of micro scans was 5 for MS, and the in-source trapping (IST) desolvation voltage was -100V. The trapping gas flow was at 7.5. The resolution setting for MS was 6250 (m/z 400). The AGC target was 1E6 for MS. The maximum injection time was 200 ms for MS. The mass range for MS scans was 2000-16000 m/z. For each protein complex sample extracted from the log or stationary phase, the 25 nL (~125 ng) *E. coli* sample was injected into the separation capillary for nCZE-MS quantification analysis. A 75-minute CZE separation with 30 kV applied at the BGE end, and 1 psi was applied at the same time, followed by 20 psi pressure applied for 10 min to flush the capillary.

In-source CID

For the in-source CID experiment, the instrument setup was the same as above, except that the automated CE operation was performed using an ECE-001 CE autosampler from CMP Scientific.

The transfer capillary temperature was 250 ^{o}C, and the S-lens RF level was 200. The number of micro scans was 5 for MS. The in-source trapping (IST) desolvation voltage was -100V, and the in-source CID was 200 eV. The trapping gas flow was at 7.5. The resolution setting for MS was 6250 (m/z 400). The AGC target was 1E6 for MS. The maximum injection time was 200 ms for MS. The mass range for MS scans was 1500-15000 m/z. The *E. coli* sample was injected into the separation capillary for nCZE-MS with 200 mbar pressure for 18 s. For separation, 20 kV voltage and 130 mbar were applied to the injection end for 40 min, followed by 800 mbar pressure applied for 15 min to flush the capillary.

Denaturing CZE-MS for top-down proteomics

The protein complex extracted from the *E. coli* cell in the log phase was subjected to the denatured top-down proteomics experiment, which is performed using a Beckman CESI8000 Plus capillary electrophoresis system coupled to a Thermo Exploris 480 Orbitrap mass spectrometer via the EMASS-II interface.

For CZE separation, a high voltage of 30 kV was applied for 70 minutes, including 10 minutes of flushing method with 30 kV and 10 psi forward. Sample injection was carried out using 5 psi air pressure, with an injection volume of 25 nL, calculated based on Poiseuille's law. The background electrolyte (BGE) consisted of 5% acetic acid, while the sheath liquid contained 0.2% formic acid and 10% methanol (v/v).

Low-high mode was employed for the TDP analysis. The transfer capillary temperature was 320 ^{o}C, and the S-lens RF level was 60. MS1 acquisition was performed at a resolution of 7,500 with AGC (automatic gain control) target as 3E6 and five microscans. The maximum injection time was set to auto, with a minimum intensity threshold of 1E4. The mass range for MS scans was 600-3000 m/z. Charge states ranging from +5 to +60, along with undetermined charge states, were included. Dynamic exclusion was enabled after three occurrences, with an exclusion duration of 30 seconds. The six most intense ions were selected for MS/MS, using an isolation window of 4 m/z. Fragmentation was performed using stepped higher-energy collisional dissociation (HCD) at 25%, 35%, and 45%. The mass range for MS scans was 200-2000 m/z. MS2 acquisition was

conducted at a resolution of 480,000, with AGC target of 1E5 and a single microscan. The maximum injection time remained in auto mode.

Data analysis

For the native CZE-MS data obtained from the native quantification experiment and in-source CID experiment, all the mass spectra were first averaged for a time window of every 30 s, followed by inputting the data into UniDec[4]. Only peaks with S/N better than 10 were analyzed. Most of the settings of UniDec analysis were at default values except for applying the 'Automatic m/z Peak Width' and the 'Suppress Artifacts' with 'Some' and 'Lots', respectively. Next, the successive charge state distribution of proteoforms/protein complexes was manually checked to ensure the correct distribution. Finally, we calculated the mass and the standard deviation of the proteoform/protein complex by ESIProt[5] based on the m/z of the successive charge states from UniDec. The m/z values used for ESIProt and the standard deviations of the deconvolved masses are listed in **Supporting Information II**. We ensured that the data from ESIProt and UniDec agreed with each other for all the proteoforms/protein complexes in **Supporting Information II**.

For the TDP experiment data analysis, the UniDec was employed for the MS^1 spectral deconvolution. By matching the observed masses with those listed in UniProt, the proteoform candidates were determined. Meanwhile, the raw file was converted to mzML files by MSConvert[6] using a peak-picking algorithm. Then, the spectral deconvolution was performed using Top-down mass spectrometry Feature Detection (TopFD, version 1.7.3)[7] with the default parameters except that the maximum charge setting as 100, the maximum mass as 100000 Da, the minimum scan number as 1, and enable Missing MS1 spectra, with the msalign file containing fragment ions obtained.

To identify the target proteoform among the candidates, ProSight Lite software[8] was employed. The precursor mass obtained from MS^1 spectral deconvolution and the corresponding fragment ion masses extracted from the msalign file were used as experimental input data. The amino acid sequences of the candidate proteoforms retrieved from UniProt served as the candidate sequences.

After entering the fragment ions and protein sequences, the average precursor mass and M (neutral) fragment mass modes were selected, with the fragment matching tolerance set to 20 ppm. Among

the candidate proteoforms, the one exhibiting the highest number of matching fragment ions was considered the target proteoform.

Statistical analysis

For the CZE-MS-based quantification experiment, the quantification was performed using peak intensities extracted from the native MS^1 spectra across runs. For subsequent statistical analysis, only protein complexes detected in at least two of the three biological replicates in both growth phases were included. Firstly, the intensity result was subjected to Perseus software (v2.0.3.0) for the missing value imputation[9]. Then, the quantitative proteomics data were analyzed in an R script (v4.3) using a custom pipeline. Protein intensities were $\log_2$-transformed and median-normalized across samples. Differential abundance between the two growth phases was assessed using both the limma moderated t-test and the nonparametric Wilcoxon rank-sum test. Log_2 fold changes and 95% confidence intervals were estimated by bootstrap resampling (1,000 iterations), with optional permutation-based p-value estimation. Multiple testing correction was applied using the Benjamini–Hochberg FDR method, and results were visualized as a volcano plot using ggplot2. The obtained proteoforms/complexoforms differentially expressed between the two growth phases were further validated by the GraphPad Prism 10.0. Comparisons between the two groups were performed using unpaired Student's t-tests. Welch's correction was applied to adjust for differences in data distributions, with p-values <0.05 were considered statistically significant.

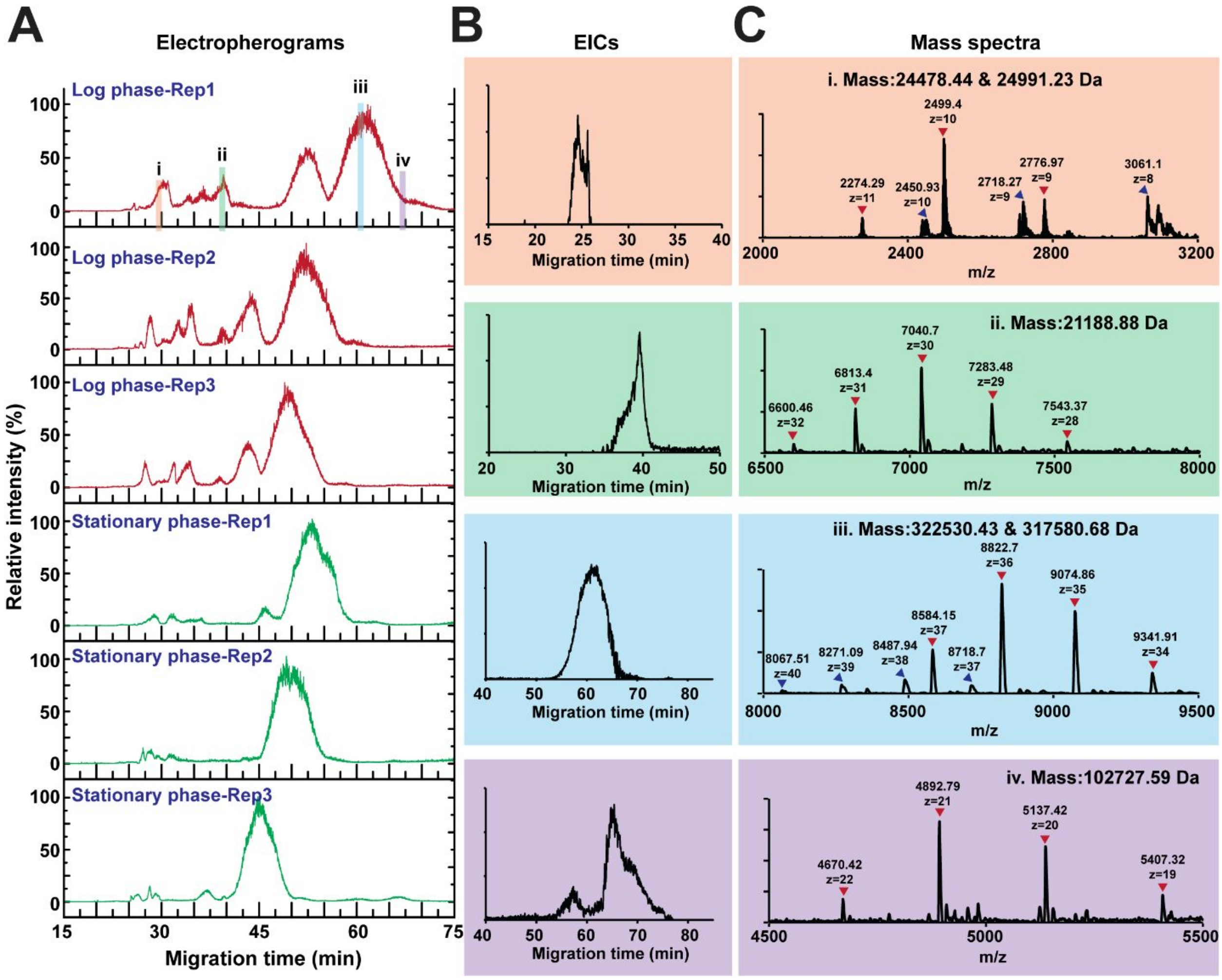

Figure S1. Representative nCZE-MS analysis of endogenous complexoforms from *E. coli* cell lysate. (A) The electropherograms of the whole *E. coli* cell lysate obtained from log and stationary phases in biological triplicates, respectively. The electropherograms are raw data without migration time alignment. (B) Extracted ion electropherograms (EICs) of example proteoforms/complexoforms in peaks i, ii, iii, and iv (from top to bottom), shown in (A). The mass tolerance is set to 500 ppm, and Gaussian smoothing was enabled at 7 points. (C) Mass spectra of proteoforms/complexoforms corresponding to the four peaks (i, ii, iii, and iv) in (A), detected from one run of *E.coli* cell lysate in the log phase. The charge states and deconvolved mass of each proteoform/complexoform are labeled.

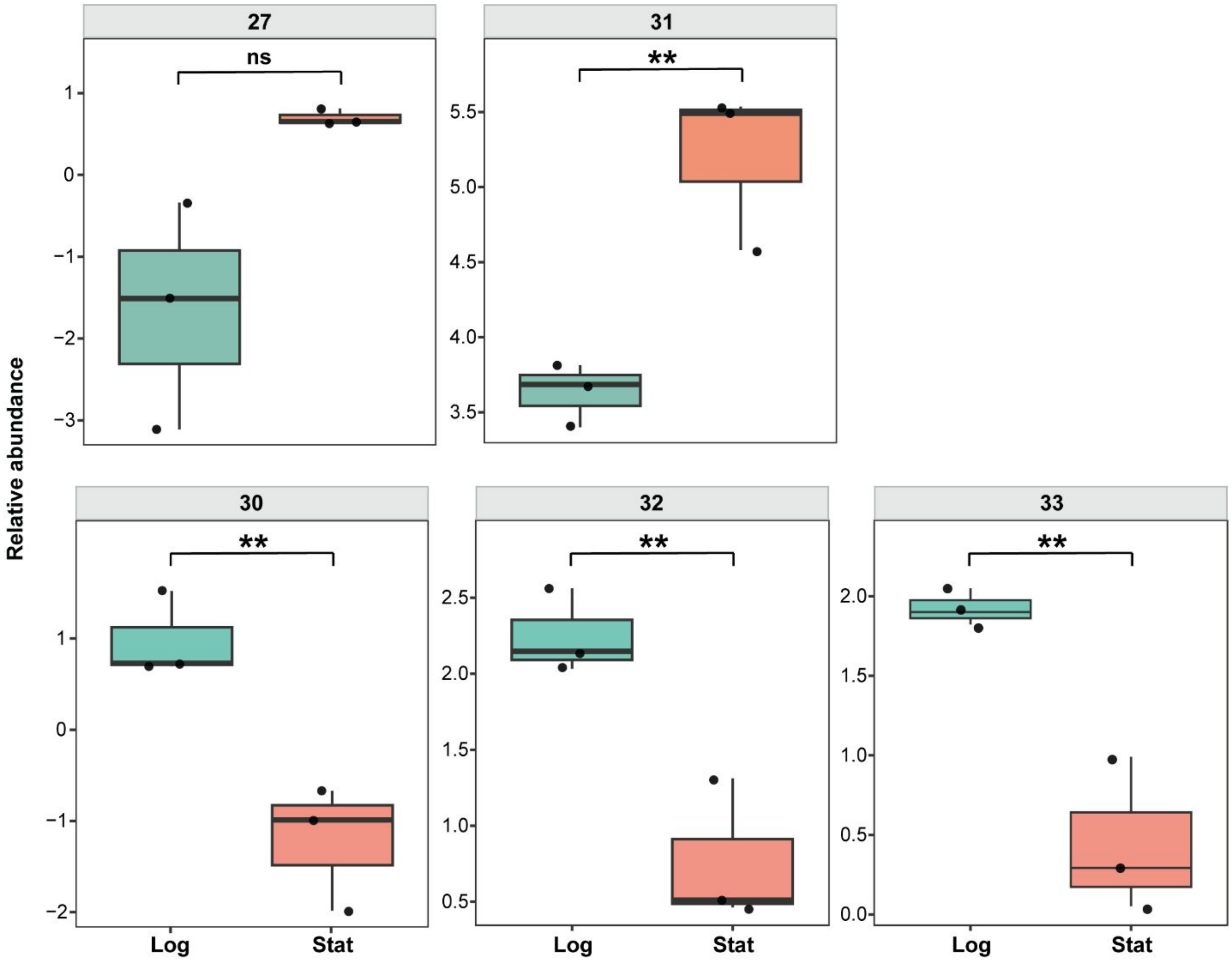

Figure S2. Box plots of relative abundance for significantly altered complexoforms obtained from **Figure 2A**. The unpaired Welch's test was conducted to compare the abundance difference of complexoforms between two growth phases. ** denotes p value ≤ 0.01 and ns denotes not significant.

Table S1. The repeatability of migration time and peak intensity was evaluated for selected complexoform peaks (corresponding to those shown in **Figure S1**) in the *E. coli* stationary phase sample using nCZE-MS, with migration times aligned to the most abundant peak.

	**Migration Time (min)**				**Peak Intensity**			
**Run \ complexoform**	**i**	**ii**	**iii**	**iv**	**i**	**ii**	**iii**	**iv**
Stationary-Rep1	23.67	28.19	43.32	49.47	4.05e3	5.56e4	4.29e4	2.36e4
Stationary-Rep2	23.82	28.13	44.07	48.78	3.13e3	4.44e4	5.21e4	2.14e4
Stationary-Rep3	22.46	29.48	43.42	47.56	4.71e3	3.42e4	4.26e4	1.35e4
**RSD (%)**	3.2	2.67	0.94	1.99	20	24	12	27

References

(1) Sadeghi, S. A.; Fang, F.; Tabatabaeian Nimavard, R.; Wang, Q.; Zhu, G.; Saei, A. A.; Sun, L.; Mahmoudi, M. Mass Spectrometry-Based Top-down Proteomics for Proteoform Profiling of Protein Coronas. *Nat Protoc* **2025**, 1–34.

(2) Sun, L.; Zhu, G.; Zhao, Y.; Yan, X.; Mou, S.; Dovichi, N. J. Ultrasensitive and Fast Bottom-up Analysis of Femtogram Amounts of Complex Proteome Digests. *Angew. Chem., Int. Ed.* **2013**, *52* (51), 13661–13664.

(3) Sun, L.; Zhu, G.; Zhang, Z.; Mou, S.; Dovichi, N. J. Third-Generation Electrokinetically Pumped Sheath-Flow Nanospray Interface with Improved Stability and Sensitivity for Automated Capillary Zone Electrophoresis–Mass Spectrometry Analysis of Complex Proteome Digests. *J. Proteome Res.* **2015**, *14* (5), 2312–2321.

(4) Marty, M. T.; Baldwin, A. J.; Marklund, E. G.; Hochberg, G. K. A.; Benesch, J. L. P.; Robinson, C. V. Bayesian Deconvolution of Mass and Ion Mobility Spectra: From Binary Interactions to Polydisperse Ensembles. *Anal. Chem.* **2015**, *87* (8), 4370–4376.

(5) Winkler, R. ESIprot: A Universal Tool for Charge State Determination and Molecular Weight Calculation of Proteins from Electrospray Ionization Mass Spectrometry Data. *Rapid Commun Mass Spectrom* **2010**, *24* (3), 285–294.

(6) Kessner, D.; Chambers, M.; Burke, R.; Agus, D.; Mallick, P. ProteoWizard: Open Source Software for Rapid Proteomics Tools Development. *Bioinformatics* **2008**, *24* (21), 2534–2536.

(7) Basharat, A. R.; Zang, Y.; Sun, L.; Liu, X. TopFD: A Proteoform Feature Detection Tool for Top–Down Proteomics. *Anal Chem* **2023**, *95* (21), 8189–8196.

(8) Fellers, R. T.; Greer, J. B.; Early, B. P.; Yu, X.; LeDuc, R. D.; Kelleher, N. L.; Thomas, P. M. ProSight Lite: Graphical Software to Analyze Top-Down Mass Spectrometry Data. *Proteomics* **2015**, *15* (7), 1235–1238.

(9) Tyanova, S.; Temu, T.; Sinitcyn, P.; Carlson, A.; Hein, M. Y.; Geiger, T.; Mann, M.; Cox, J. The Perseus Computational Platform for Comprehensive Analysis of (Prote)Omics Data. *Nat Methods* **2016**, *13* (9), 731–740.
